# Toll‐like receptor 4 promoter polymorphisms in a familial mediterranean fever patient with asymptomatic bacteriuria

**DOI:** 10.1002/ccr3.1941

**Published:** 2018-11-25

**Authors:** Shinichiro Kawamoto, Ryota Masutani, Keiji Kurata, Etsuko Shima, Yoshinori Iwatani, Takayuki Takubo

**Affiliations:** ^1^ Department of Transfusion Medicine and Cell Therapy Kobe University Hospital Hyogo Japan; ^2^ Department of Central Clinical Laboratory Osaka Medical College Hospital Osaka Japan; ^3^ Department of Biomedical Informatics, Division of Health Sciences Osaka University Graduate School of Medicine Osaka Japan; ^4^ Department of Medical Oncology/Hematology Kobe University Hospital Hyogo Japan; ^5^ Department of General Medicine Kobe Central Hospital Hyogo Japan; ^6^ Japanese Red Cross Society Osaka Japan

**Keywords:** asymptomatic bacteriuria, familial Mediterranean fever, outer membrane vesicles, SNPs, TLR4

## Abstract

A periodic fever, due to inherited inflammatory disorders, can be misdiagnosed as a common infection, when a possible pathogen is detected from a patient. *TLR4* SNPs that are responsible for asymptomatic bacteriuria might disturb the pathophysiology of familial Mediterranean fever without *MEFV* mutations.

## 1 INTRODUCTION

At the onset of a periodic fever, a persistent infection is suspected first as the cause, and the patient is examined extensively to identify the focus and pathogen. However, candidate pathogens are not usually detectable in samples obtained from a pleural effusion or ascites in cases of familial Mediterranean fever (FMF), despite the presence of an elevated white blood cell and high C‐reactive protein levels. After excluding autoimmune diseases, the observation of an aseptic inflammatory condition with a spontaneous recovery might lead the physician to suspect a diagnosis of FMF after a long clinical course.^1^

Asymptomatic bacteriuria (ABU) is frequently observed in young women and is a strong predictor of symptomatic urinary tract infections (UTIs).^2^ As it might not be rare for both a UTI and FMF to coincide in a female patient, patients with periodic fever are therefore managed assuming this possibility in an endemic area.^3^ However, if pathogens are detected in urine samples from patients with recurrent fever, a diagnosis of FMF is extremely difficult without careful attention, especially in a nonendemic area.

FMF patients are characterized by a hyperactive immune response, owing to a mutation in the Mediterranean fever (*MEFV*) gene, which encodes the pyrin protein. Pyrin is a suppressor of the inflammasome that activates caspase‐1, leading to conversion of pro‐interleukin (IL)‐1b to its active form, IL‐1b.^4^

Meanwhile, toll‐like receptor (TLR) 4 also plays critical roles in the immune response to exogenous pathogens by the induction of pro‐IL‐1 transcription. Thus, TLR4 collaborates with pyrin for the production of mature IL‐1. The relationship between the aberrant immune response and*TLR* single‐nucleotide polymorphisms (SNPs) has been reported in several infections, including UTIs.^5^, ^6^ However, the relationship between *TLR4* SNPs and UTIs in FMF patients has not been fully elucidated.^7^

In the present study, we investigated *TLR4 *promotor SNPs in a periodic fever patient without frequent *MEFV* mutations to determine their role in ABU. Then, we review ABU as a common disease in sexually active women. Based on these facts, we suggest a mechanism for periodic fever that is triggered by the release of bacterial vesicles in response to outer stimuli during the menstrual period, which might be another cause of FMF.

## 2 CASE REPORT

A female patient had undergone a gastrectomy for gastric cancer when she was 29 years old. Soon after the operation, symptoms developed, including a high‐grade fever, back pain, lower abdominal pain, the feeling of residual urine, and left knee joint pain. The symptoms recurred every month for a few days, primarily around the time of menstruation. The patient was examined extensively by computed tomography, esophagogastroduodenoscopy, and colonoscopy, but no abnormal findings were revealed. As the symptoms were also able to be induced by a UTI, with the exception of the knee joint pain, a diagnosis of pyelonephritis was made by a urologist, after a voiding cystography without any abnormal findings. Thereafter, she was treated with antibiotics and nonsteroidal anti‐inflammatory drugs (NSAIDs) for eight years. She also felt fatigue and experienced appetite loss for a long duration, even during afebrile periods. In recent years, she has tended to stay indoors at all times.

When the patient was 37 years old, she was referred to our hospital for iron deficiency anemia and dysgeusia after the administration of ferric medicine; these conditions were possibly caused by the appetite loss following the periodic fever. On the basis of precise history‐taking and laboratory examinations, including elevation of serum amyloid A (SAA), we suspected FMF (Table 1).

**Table 1:** Laboratory examinations

Peripheral blood and biochemical parameters					
WBC	6.9	×10^9^/L	AST	18	IU/L
Seg	69.5	％	ALT	13	IU/L
Eo	4.0	％	LDH	145	IU/L
Ba	0.5	％	ALP	765	IU/L
Mo	7.5	％	γ‐GTP	19	IU/L
Ly	17.5	％	T.Bil	0.6	mg/dL
At‐Ly	1.0	％	BUN	10.9	mg/dL
RBC	5.20	×10^12^/L	Cr	0.54	mg/dL
Hb	139	g/L	Na	139	mEq/L
Ht	41.9	％	Cl	105	mEq/L
MCV	80.6	fL	K	3.9	mEq/L
MCH	26.7	pg	Ca	8.2	mg/dL
MCHC	33.2	％	P	2.7	mg/dL
PLT	243	×10^9^/L	CRP	0.11	mg/dL
			SAA	23.4	µg/mL
			ANA	1:40	
Urinalysis and two consecutive midstream urine cultures					
Protein	(−)	RBC	1‐4/HPF	Bacterial culture 1	
Urobilinogen	(±)	WBC	≥100/HPF	*Klebsiella pneumonia*	
Sugar	(−)	Squamous cell	5‐9/HPF		
Acetone	(−)	Hyaline cast	˂1/LPF	Bacterial culture 2	
Bilirubin	(−)	Bacteria	˃10 000/μL	*Klebsiella pneumonia*	
pH	7				

ANA, antinuclear antibody; SAA, serum amyloid A.

Colchicine treatment (0.5 mg/d) was initiated, according to the diagnostic criteria for FMF.^3^ The symptoms disappeared completely without treatment with antibiotics or NSAIDs. Despite the improvement, *Klebsiella pneumoniae* (*K. pneumoniae*) continued to be reproducibly detected at a high titer in consecutive urine cultures before and during menstruation (Table 1).

After informed consent was obtained from the patient, DNA sequencing was performed. Recurrent *MEFV *mutations were not detected in exons 1, 2, 3, or 10 (data not shown).

**Figure 1:**
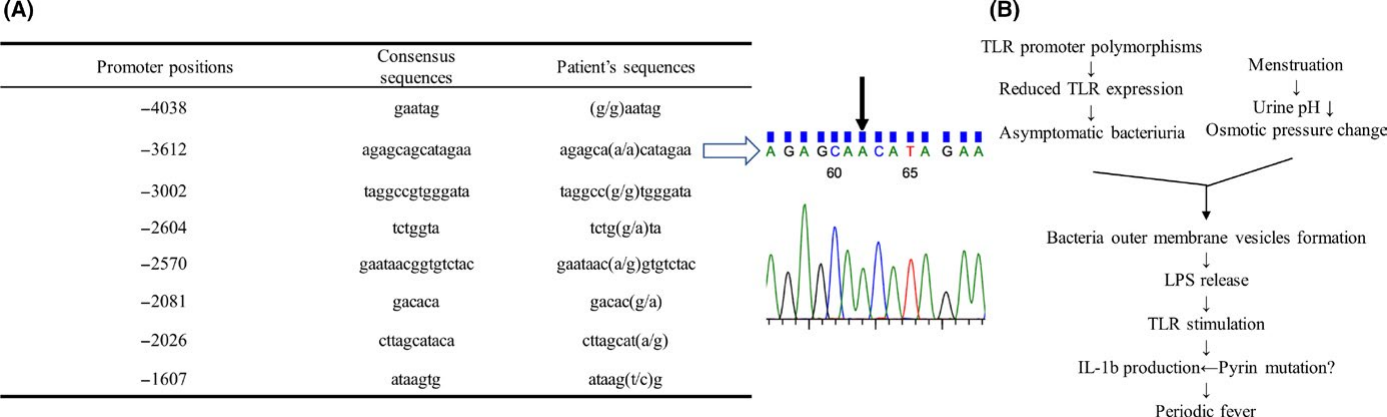
A, *TLR4* promoter consensus DNA sequences and SNPs in the present case at the indicated positions. Closed arrow indicates the rare homozygote revealed by direct sequencing at −3612. B, Schema of periodic fever induction that is possibly affected by *TLR4* polymorphisms and menstruation through bacteria colonization in the present case. Abbreviation: LPS, lipopolysaccharide

To further evaluate the deteriorated immune response in the patient, we analyzed SNPs in the*TLR2* exon and the promoters of *TLR4*.^8^, ^9^ The *TLR2 *exon of the patient showed a G/G allele, corresponding to Arg753Arg, which is a common genotype in the non‐UTI control (data not shown). On the other hand, the *TLR4* promoter region included six of the eight SNPs that had been found in Swedish patients with ABU (Figure 1A).

## 3 DISCUSSION

It takes a longer time period to diagnose FMF from the onset of a periodic fever in a nonendemic area than in an endemic area.^1^, ^10^ The present patient had been treated for recurrent UTIs for eight years, probably due to the continuous detection of *K. pneumoniae*. Although there are no specific laboratory tests, except for *MEFV* gene analysis, designed to diagnose FMF, mutations are not detected in some FMF cases.^11^ In such cases, the diagnosis of FMF is mostly dependent on the clinical manifestations listed in criteria (Table 2 from Ref. 3). The current case showed symptoms, such as pleuritis‐like back pain and monoarthritic (knee) pain that are included in the major criteria. In addition, the patient also showed localized abdominal pain that required extensive examinations. It is noteworthy that severe, requiring bed rest, which is listed in supportive criteria 4, improved dramatically soon after the administration of colchicine. As *K. pneumoniae* was detected even after the improvement, we suspected an existence of genetic variation that causes disturbed immune response leading to ABU and periodic fever. Instead of *MEFV* mutations, we identified multiple SNPs in the *TLR4* promoter of the patient.

**Table 2:** Criteria set for diagnosis of familial Mediterranean fever suggested by Livneh et al^a^

Major criteria
Typical attacks
1. Peritonitis (generalized)
2. Pleuritis (unilateral) or pericarditis
3. Monarthritis (hip, knee, ankle)
4. Fever alone
Minor criteria
1‐3. Incomplete attacks involving 1 or more of the following sites:
1. Abdomen
2. Chest
3. Joint
4. Exertional leg pain
5. Favorable response to colchicine
Supportive criteria
1. Family history of FMF
2. Appropriate ethnic origin
3. Age<20 years at disease onset
4‐7. Features of attacks
4. Sever, requiring bed rest
5. Spontaneous remission
6. Symptom‐free interval
7. Transient inflammatory response, with 1 or more abnormal test result(s) for white blood cell count, erythrocyte sedimentation rate, serum amyloid A, and/or fibrinogen
8. Episodic proteinuria/hematuria
9. Unproductive laparotomy or removal of white appendix
10. Consanguinity of parents

a The requirements for diagnosis of FMF are ≥1 major criteria, or ≥2 minor criteria, or 1 minor criterion plus ≥5 supportive criteria. Typical attacks are defined as recurrent (≥3 of the same type), febrile (rectal temperature of 38°C or higher), and short (lasting between 12 h and 3 d). Incomplete attacks are defined as painful and recurrent attacks that differ from typical attacks in 1 or 2 features, as follows: (a) the temperature is normal or lower than 38°C; (b) the attacks are longer or shorter than specified (but not shorter than 6 hours or longer than a week); (c) no signs of peritonitis are recorded during the abdominal attacks; (d) the abdominal attacks are localized; (e) the arthritis is in joints other than those specified. Attacks not counted

The*MEFV* gene, encoding the pyrin protein, is preferably expressed in granulocytes and is thought to inhibit excess inflammation induced by these cells.^4^ Koc et al reported that a higher frequency of *MEFV* gene mutation carriers has been observed among critically ill patients with pneumonia, UTIs, and acute pancreatitis in Turkey.^12^ Ben‐Chetrit et al also reported that there are patients with *MEFV* mutations that exhibit distinct clinical presentations not typical of FMF.^13^ Thus, patients with the *MEFV* gene show symptoms, due to the disturbed immune response, except for periodic fever.

Meanwhile, in UTI and ABU patients, SNPs in the*TLR4* promoter have been investigated, as well as in the *TLR2 *exon promoter regions.^8^, ^9^ Ragnarsdóttir et al confirmed the genotype patterns of the *TLR4* promoter in Swedish ABU patients compared to those of symptomatic UTI patients and healthy controls.^9^ The present patient carried six of the eight reported *TLR4 *promoter SNPs that did not match the consensus sequences, and the genotype pattern was almost the same as pattern VIII observed in the Swedish ABU patients, except for a rare homozygote at −3612 observed in the present case. Although it is not certain whether the genotype pattern in the present case indicates hypo‐responsiveness, the homozygote at −3612 was confirmed to reduce the *TLR4* promoter activity, leading to low TLR4 expression in vitro and frequently observed in primary pediatric ABU patients with genotype pattern V.^9^, ^14^ As it has also been confirmed that patients that carry *TLR4* genotype V show a lower neutrophil response, the reduced TLR4 expression and the insufficient response would allow FMF patients to tolerate ABU without fever.^15^

Asymptomatic UTIs are observed in approximately 5% of sexually active, healthy females and are a strong predictor of symptomatic UTIs.^2^ *Klebsiella pneumoniae* is an important causative pathogen of UTIs, next to *Escherichia coli*.^16^ The administration of antibiotic prophylaxis is not recommended, except in pregnant females.^17^ It might be reasonable that frequent *MEFV* mutations were not detected in the present case, because bacterial colonization is likely to induce a continuous fever in mutation‐positive patients. According to the guideline from the Infectious Diseases Society of America for the diagnosis and treatment of asymptomatic bacteriuria in adults, the criteria for UTI consists of the following: (a) >10 white blood cells/mm^3^ and (b) the isolation of the same bacterial strain at consecutive counts of >105 colony‐forming units/mm^3^, obtained more than 24 hours apart .^18^ The major problem in interpreting the results of urine cultures is that ~10%‐20% of females harbor Enterobacteriaceae, including *Klebsiella pneumonia*, in the vagina and periurethral area at any time. In the current case, the presence of pyuria and the isolation of a single bacterial strain, *K. pneumonia*, in consecutive cultures, even after the improvement of symptoms following colchicine administration, support the existence of an asymptomatic UTI. Although 10% to 20% of symptomatic UTIs recur after antibiotics treatment, the periodic emergence of fever in the current case differs from the manifestation of common UTIs.^2^, ^17^, ^19^

The relationship between the menstrual period and FMF attacks is controversial.^20^, ^21^ Takano et al confirmed changes in hydrogen and potassium excretion in urine during the menstrual cycle.^22^ As gram‐negative bacteria release outer membrane vesicles (OMVs) in response to several environmental stresses, such as pH and osmotic changes, menstruation might stimulate the release of OMVs from bacteria in the urinary tract.^23^ Considering that OMVs contain many components of bacteria, including lipids, proteins, lipopolysaccharides, and other molecules, they would stimulate TLR signaling,^24^ and the amount of OMVs released from ABU patients during menstrual periods might be enough to compensate for the downregulated TLR4 expression, due to the poor promoter activity. As shown in Figure 1B, we hypothesized a possible mechanism connecting the *TLR4* promoter SNPs and periodic fever triggered by menstruation in the present case. In conclusion, FMF patients, whose attacks are suspected to be triggered by the menstrual cycle, might have fewer *TLR4* promoter genotypes that allow gram‐negative bacteria to colonize the urinary tract without symptoms.

## CONFLICT OF INTEREST

None declared.

## AUTHOR CONTRIBUTION

SK: designed research and wrote the manuscript. RM and KK: performed experiments. ES: acquired data. YI and TT: contributed to research design and manuscript writing.
